# CAFÉ: a multicomponent audit and feedback intervention to improve implementation of healthy food policy in primary school canteens: a randomised controlled trial

**DOI:** 10.1186/s12966-016-0453-z

**Published:** 2016-12-05

**Authors:** Sze Lin Yoong, Nicole Nathan, Luke Wolfenden, John Wiggers, Kathryn Reilly, Christopher Oldmeadow, Rebecca Wyse, Rachel Sutherland, Tessa Delaney, Peter Butler, Lisa Janssen, Sarah Preece, Christopher M. Williams

**Affiliations:** 1 School of Medicine and Public Health, University of Newcastle, Callaghan, NSW 2308 Australia; 2 Hunter Medical Research Institute, New Lambton, NSW 2305 Australia; 3 Priority Research Centre for Health Behaviour, University of Newcastle, Callaghan, NSW 2308 Australia; 4 Hunter New England Population Health, Locked Bag 10, Wallsend, NSW 2287 Australia

**Keywords:** Nutrition, Primary schools, Nutrition policies, Implementation, Audit and feedback

## Abstract

**Background:**

The implementation of nutrition policies in schools has been recommended as a strategy to improve child dietary intake. Internationally, research suggests that the majority of schools do not implement these policies. In New South Wales (NSW), Australia, the NSW Healthy School Canteen Policy requires that school canteens prohibit the sale of ‘red’ foods (i.e. foods that are typically nutrient poor and high in energy, such as confectionary and deep-fried foods) and ‘banned’drinks (i.e. soft drinks); and that the majority of items on the menu are ‘green’ (i.e. foods that are good sources of nutrients, such fruits, vegetables and lean meats). This study examined the impact of a multicomponent audit and feedback intervention on schools’ implementation of the NSW Healthy School Canteen Policy. A secondary aim was to assess the impact of the intervention on menu composition.

**Methods:**

This study was a parallel group randomised controlled trial with 72 rural and remote primary schools (36 interventions, 36 controls) located in one region within NSW, Australia. Intervention schools received an initial face to face contact and up to four cycles of audit and feedback (consisting of a menu audit, written feedback report and telephone feedback) over a 12-month period. The primary trial outcomes were the proportion of schools with a canteen menu that had: i) no ‘red’ foods or ‘banned’ drinks; and ii) >50% ‘green’ items, as assessed via standardised menu audits undertaken by trained dietitians. For each primary outcome, between-group differences were assessed using Fisher’s exact test under an intention to treat approach.

**Results:**

There was insufficient evidence to conclude the intervention had a positive impact on the proportion of intervention schools with no ‘red’ or ‘banned’ items on their menu (RR = 2.8; 95% CI: 0.9 to 8.9; *p* = 0.0895), or on the proportion of intervention schools with more than 50% ‘green’ items (RR = 1.5; 95% CI: 0.7 to 3.2; *p* = 0.2568). These findings remained non-significant in the multiple imputation analyses. Intervention schools were significantly more likely to have a lower percentage of ‘red’ items (*p*-value: 0.007) and a higher percentage of ‘green’ items on the menu (*p*-value: 0.014). This remained statistically significant in the multiple imputation analyses for ‘red items’ (*p*-value: 0.0081) but not for ‘green’ items (*p*-value: 0.0910).

**Conclusions:**

While there was insufficient statistical evidence to suggest that this multicomponent audit and feedback intervention was effective in improving primary schools’ compliance with a healthy canteen policy, the intervention demonstrated some positive impact in reducing the availability of ‘red’ items on the menu.

**Trial registration:**

This trial was prospectively registered with the Australian New Zealand Clinical Trials Registry (ACTRN12613000543785). Registered 15th May 2013.

## Background

The 2013 Global Burden of Disease study reported that 11.3 million deaths were attributable to dietary risk factors including diets low in fruit (3.4 million deaths), vegetables (1.8 million deaths) and whole grains (2 million deaths), and diets high in sodium (3.7 million deaths) [1]. Internationally, between 1999 and 2010, a rapid increase in the consumption of foods low in nutritional value has been reported, particularly in children [2]. Studies conducted with children from high income countries also indicate inadequate consumption of fruit and vegetables, well below that recommended in national guidelines [3–5]. As dietary behaviours and patterns that are developed in childhood persist into adulthood [6], interventions to improve children’s dietary intake have been recommended to reduce the burden from dietary risk factors.

Leading health organisations including the World Health Organization (WHO) [7] and the United States (U.S.) Institute of Medicine [8] have recommended the implementation of nutrition policies in schools to modify the availability of healthy and unhealthy foods. Such recommendations are supported by systematic review evidence that these food-service based strategies improve child dietary behaviours, particularly the consumption of healthier foods and reduce the purchasing of unhealthy foods from school kiosks and vending machines [9–11]. Many countries including the U.S., UK, and Australia have introduced nutrition policies requiring schools to provide healthier food options to students [12–14].

Despite this, research suggests that the implementation of such nutrition policies is limited. For example in the U.S., the 2012 School Health Policies and Practices Study found that more than half of the surveyed schools across all states sold high energy, nutrient poor foods, despite nutrition policies restricting the availability of such foods [15]. A national study of 263 Australian primary and high schools found low rates of implementation of healthy nutrition policies in schools canteens across all states (prevalence of between 5 and 35%) except for Western Australia (where 62% were implementing the policy) [14]. A study of 318 New Zealand schools found that 66% reported implementing changes after the introduction of the 2008 Guidelines on Food and Nutrition that required schools to provide only healthy menu options [16]. Of those schools that made changes, 12% reported modifying their food policies, 5.7% reported removing fruit juices and sweetened drinks, and 5.7% introduced healthier food choices [16]. Differences in the implementation of nutrition policies are also apparent by a number of school factors. In Australia for example, rural school canteens had significantly poorer implementation of a mandatory school canteen policy [17]. While such findings suggest that additional efforts to support policy implementation in rural schools may be required [17], providing such support across a population of schools that are geographically dispersed represents a considerable challenge to health policy makers and practitioners.

A number of barriers to the implementation of canteen policies are frequently reported, including inadequate resources and a lack of knowledge and skills to appropriately identify and classify healthy foods consistent with policy requirements [18]. Interventions that address such barriers are required to facilitate policy implementation and maximise the potential benefits to child nutrition that the policy was intended to deliver to the community. However, an updated Agency for Health and Quality Research review published in 2012 found only one quasi-experimental controlled trial that sought to improve nutrition policy implementation in schools [19]. This trial involved the provision of training, financial support for staff relief, and evidence-based resources to eight elementary schools located in a low socioeconomic region in U.S. [20]. The study found no significant difference in the nutritional content of school meals provided between intervention and control schools post-intervention, providing limited guidance to support future implementation of nutrition policies in schools [20].

Applied theoretical frameworks for implementation science suggest that audit and feedback may be an effective strategy in overcoming the primary impediments reported by school staff (lack of knowledge and skill in food classification) in nutrition policy implementation [21]. The Cochrane Effective Practice of Care review group defines audit and feedback as “a summary of an individual’s performance over a period of time, provided in written, electronic or verbal format” [22]. Similarly, evidence from observational studies in schools suggest that audit and feedback can effectively support school curriculum planning [23]; and improve teaching quality [24] and student learning outcomes [25]. Furthermore, systematic review evidence from health care settings consistently reports that audit and feedback is an effective strategy to support guideline implementation and can maintain its effectiveness when delivered across non-face to face modalities (including telephone and written) [26, 27]. Importantly, non-face to face modalities represent particularly attractive methods of providing support in rural and remote locations and overcome significant geographic restrictions associated with the provision of face to face support.

## Objectives

Given the limited available literature to support healthy canteen policy implementation in schools, and the potential of audit and feedback as a strategy to support implementation, particularly in rural and remote schools, this study (the Canteen Audit and Feedback Effectiveness study – CAFÉ) aimed to assess the impact of an audit and feedback intervention on improving the proportion of rural and remote schools that were compliant with the NSW Healthy School Canteen Policy. In Australian primary schools, food and beverages are typically pre-packaged or made on site and sold over the counter for children to eat at lunch and/or recess (a shorter break time either before or after lunch). All foods sold in the canteen are listed on the menu and no standard meal service is provided by the school. As such, the policy focuses specifically on the types of foods that are made available for sale via school canteens and vending machines. The policy requires that school canteens prohibit the sale of ‘red’ foods (i.e. foods that are typically nutrient poor and high in energy, such as confectionary and deep-fried foods), limit the sale of ‘amber’ foods (i.e. foods considered to have some nutritional value, but may contribute to excess energy, such as processed meats and full fat dairy) and that the majority of foods sold on the menu are ‘green’ (i.e. foods that are good sources of nutrients, such fruit, vegetables and lean meats).

A secondary aim was to assess the impact of the intervention on menu composition (i.e. the percentage of ‘red’, ‘amber’ and ‘green’ items on the menu). The study also assessed the acceptability of the intervention components, schools’ receipt of other support to improve policy compliance, and examined the association between the number of audit and feedback cycles provided and canteen compliance to the NSW Healthy School Canteen Policy.

## Methods

### Trial registration

This trial was prospectively registered with the Australian New Zealand Clinical Trials Registry (ANZCTR) (ACTRN12613000543785). A detailed protocol for this trial has been previously published [28].

### Design and setting

CAFÉ was a single-blinded, parallel group randomised controlled trial conducted in the Hunter New England Local Health District of New South Wales, Australia. The Hunter New England Local Health District covers a large geographic region (more than 130,000km^2^) and consists of a socioeconomically and demographically diverse population of approximately 112,000 children aged 5–12 years.

Only the outcome assessors, which included CATI Interviewers and dietitians undertaking menu audits, were blinded to the intervention, as the nature of the intervention meant that schools were unable to be blinded to group allocation.

#### Participants and recruitment

All primary schools (i.e. those enrolling children aged 5–12 years) in rural or remote areas within the study region (i.e. school postcode was classed as corresponding to an outer regional, rural or remote area according to the Accessibility/Remoteness Index of Australia (ARIA) score [29]), that had previously reported having a canteen (in a 2012 telephone interview conducted by the research team with school Principals) were assessed for eligibility to participate in the study (*n* = 150). Schools were eligible to participate if they: i) had a canteen open at least one day per week; AND ii) were not compliant with the NSW Healthy School Canteen Policy, defined as either having at least one canteen menu item restricted for sale (‘red’ or ‘banned’ item) or having less than 50% of menu items classified as healthy (‘green’ items) (based on dietitian assessment of the supplied canteen menu). Schools enrolling both primary and secondary students (i.e. central schools) and schools catering exclusively for children requiring specialist care were excluded.

### Randomisation and allocation

All eligible schools were randomised into the intervention or control group in a 1:1 ratio by an independent investigator using a computer-generated randomisation schedule in Microsoft Excel.

### Intervention

#### Policy context

As part of the New South Wales obesity prevention strategy [30], in 2005 the State government introduced the NSW Healthy School Canteen Policy (henceforth known as ‘Fresh Tastes @ School’) [31]. The policy was based on the 2003 Australian Dietary Guidelines and utilises a traffic light system to classify menu items as ‘red’, ‘amber’ or ‘green’ based on their nutritional profile (including energy, saturated fat, and/or salt). ‘Red’ items are typically nutrient poor, high energy foods; ‘amber’ items are considered to have some nutritional value, however if consumed in large amounts can contribute to excess energy intake, and ‘green’ items are those that are considered to be good sources of nutrients, such as fruit, vegetables and lean meats. Sugar-sweetened drinks that are >300 kJ/serve and/or have >100mg of sodium/serve are classed as ‘banned’ items, and are not to be sold in school canteens. The ‘Fresh Tastes @ School’ policy requires that schools: i) provide primarily ‘green’ items (>50% of the menu) and ii) restrict the sale of ‘red’ foods and remove ‘banned’ drinks. Government primary schools are mandated to implement the policy, while implementation amongst non-government schools is strongly encouraged. Supporting the implementation of ‘Fresh Tastes @ School’ is the responsibility of local health promotion service delivery teams across the state. The intervention (consisting of up to four menu audits together with verbal and/or written feedback) was delivered by Health Promotion Officers as part of routine service delivery in the study region. It specifically involved the provision of implementation support to canteen managers to exclude ‘red’ foods and ‘banned’ drinks and to increase the proportion of ‘green’ items provided on menus.

### Data collection procedures

At baseline (Feb–Oct 2013) and follow-up (Sept 2014–Jan 2015), Canteen Managers and school Principals completed a Computer Assisted Telephone Interview (CATI). The CATI was administered by trained interviewers using a standardised script and assessed items related to school and canteen characteristics, perceived usefulness of the intervention, and exposure to other nutrition interventions. As part of the CATI, Canteen Managers were also asked to supply their current canteen menu and participate in follow-up telephone calls from dietitians to collect additional information required to assess menu compliance. Menu audits undertaken by two trained dietitians blinded to group allocation (described in detail in a previous publication [32]) were used to assess the primary trial outcome (compliance with the ‘Fresh Tastes @ School’ policy). If additional information was required to classify a menu item according to the policy, dietitians collected additional product information from Canteen Managers via phone or email using a standard data collection template. A percentage agreement of over 90% was achieved between the menu reviews of two independent dietitians, in relation to policy compliance [32].

### Theoretical framework

The selection of specific Canteen Manager behaviour change techniques was based on Control Theory, which has previously been applied to audit and feedback interventions in healthcare settings [33]. The theory suggests that the key behaviour change techniques to target knowledge gaps and skill barriers include: feedback on current performance, goal setting to allow comparison between current and target performance, and development of action plans to support target attainment [33].

### Intervention design

The main intervention components included a menu audit, and subsequent provision of feedback via a written report and telephone call. This cycle of audit and feedback was delivered each school term (four times) over a 12-month period. The specific intervention components related to the frequency of feedback and the design of the feedback report were based on systematic review evidence of effective components of audit and feedback interventions in health care settings [26]. This included provision of: i) feedback more than once in a 12-month period (up to 4 times); ii) feedback in both written and verbal formats; iii) feedback from a reputable source (e.g. health promotion team known to schools); iv) specific feedback on how to address key targets for change (e.g. the removal of ‘red’ foods and ‘banned’ drinks, and suggestions for alternatives, as well as strategies to improve the provision of ‘green’ items); and v) efforts to obtain institutional support for the delivery of feedback (e.g. holding an initial face to face meeting with the Principal and Canteen Manager to obtain their support, where possible). The specific number of menu audits, feedback reports and calls provided was tailored depending on each school’s compliance with the guidelines and whether menu changes had occurred between the previous and planned menu audit; and the responsiveness of the Canteen Manager to the feedback (e.g. whether they declined to take feedback calls).

### Menu audit

A dietitian who was blind to group allocation and not involved in the delivery of the intervention undertook audits of the canteen menus using a standardised template based on the ‘Fresh Tastes @ School’ policy. Canteen Managers were requested to provide a copy of their menu and additional information needed to assess menu compliance with the ‘Fresh Tastes @ School’ policy (as described above). Additional phone contact was made by the dietitian as needed to obtain all necessary information to classify menu items as ‘red’, ‘banned’, ‘amber’ and ‘green’ according to the policy. Subsequent menu audits were planned for each term (four over a 12-month period) with verbal and written feedback (described below) provided after each audit.

### Feedback report

Dietitians developed a standard feedback report template which summarised whether or not the school menu complied with the ‘Fresh Tastes @ School’ policy. The feedback report was delivered via email or mail by a member of the health promotion team, depending on individual school preference. The report graphically displayed the proportion of ‘red’, ‘banned’, ‘amber’ and ‘green’ items on the menu and outlined the school’s compliance with the ‘Fresh Tastes @ School’ policy. Specific suggestions on how to change canteen menus to meet the requirements of the ‘Fresh Tastes @ School’ policy were provided including: healthy recipes, ideas about how to increase the number of ‘green’ items on their menu, and alternative food options to replace specific ‘red’ foods or ‘banned’ drinks. The health promotion staff also provided other useful resources based on Canteen Managers’ reported requirements, as assessed during the feedback calls (described below in ‘Resources’).

### Feedback calls

During the initial feedback call, the Health Promotion Officer reiterated the purpose of the report, discussed the results, clarified any unclear components, supported the Canteen Manager to undertake a goal setting process to identify key areas for improvement in order to improve compliance with the ‘Fresh Tastes @ School’ policy, and developed an action plan to overcome existing barriers to policy compliance. In all subsequent calls (two to four), the Health Promotion Officer tailored the discussion to the needs of the Canteen Manager based on previous contact; and monitored their actions and progress toward their goals, set new goals where required, or monitored compliance. Where possible, the same Health Promotion Officer provided support to the same school throughout the intervention period. Two of the five Health Promotion Officers had qualifications in nutrition and dietetics, however all support officers received the same training in implementing strategies to support organisational change processes and intervention delivery.

### Resources

All intervention schools were provided with ‘Fresh Tastes @ School’ resources (including a Canteen Menu Planning guide, recipes, and a manual on financial management and food safety), healthy food guidelines, a menu planning template, sample policies and menus, pricing guides and a local suppliers buyer’s guide which lists foods that meet the ‘amber’ and ‘green’ criteria, developed by a state nutrition agency which provides canteen support to their member schools (Healthy Kids Association).

### Control group

Schools in the control group received the standard offer of professional development opportunities provided through the NSW Government’s *Live Life Well @ School* initiative (https://www.healthykids.nsw.gov.au/teachers-childcare/live-life-well-@-school/workshops-and-conferences.aspx). This initiative supports schools to implement whole of school strategies to promote healthy eating and physical activity programs for students. As part of this initiative, a two-day training program is offered that specifically targeted teachers’ skills, knowledge and confidence in teaching nutrition and physical activity as part of the school syllabus. This training does not comprehensively cover the implementation of the ‘Fresh Tastes @ School’ policy and Canteen Managers are unable to access such training opportunities. No support to implement the Fresh Tastes@ School was specifically provided by the local health promotion unit.

### Primary outcomes

#### Compliance to the ‘Fresh Tastes @ School’ Policy

The primary trial outcomes were the proportion of schools having a canteen menu that: i) did not contain any ‘red’ foods or ‘banned’ drinks; and ii) contained >50% ‘green’ items as specified by the ‘Fresh Tastes @ School’ policy. The dietitians classified all food and beverage menu items as either ‘green’, ‘amber’, ‘red’ or ‘banned’ according to the ‘Fresh Tastes @ School’ policy (as described above). Discrepancies in product classification between dietitians were resolved through discussion, or if agreement could not be reached, a third independent dietitian determined the classification.

### Secondary outcomes

#### Menu composition – percentage of ‘green’ and ‘red’ foods

Menu composition was determined by tallying all items on the menu to produce a count of the total number of menu items, and determining the percentage of ‘green’, ‘amber’, and ‘red’ items on the menu.

### Process measures

#### Perceived usefulness of intervention

During the follow-up CATI, Canteen Managers in the intervention group reported on a 5-point Likert scale ranging from ‘not helpful’ to ‘extremely helpful’ the extent to which they found each intervention component (email contact, menu audit and feedback report, and telephone support) helpful to support implementation of the ‘Fresh Tastes @ School’ policy:

#### Receipt of other support to implement the policy

Principals and Canteen Managers reported during the follow-up CATI whether they had received any support to implement the ‘Fresh Tastes @ School’ policy, and the sources of such support. They were also asked if they were currently a member of the Healthy Kids Association. The Healthy Kids Association is a non-government organisation funded to support member schools to implement ‘Fresh Tastes @ School’ through the provision of resources, telephone support and two menu feedback cycle per year.

#### Number of feedback reports and calls provided

Project staff used a spreadsheet to centrally record the date and content of each menu audit for each intervention school. They also recorded the date that the feedback report was posted and the date of the feedback call to schools, as well as the type and number of hard copy resources provided. At the end of the intervention period, a Research Assistant not involved with intervention delivery, counted the number of menu audits, feedback reports and feedback calls provided to each school.

### Statistical analyses

All analyses were performed using SAS 9.3 (SAS Institute Inc., Cary, NC) by an independent statistician not involved in the trial and not co-located with the research team. School and canteen characteristics are presented for intervention and control groups using means and standard deviations for continuous variables and counts and percentages for categorical variables. School and canteen characteristics were compared between schools that did and did not complete follow-up menu audits using Pearson Chi-square tests or student’s t-tests. The primary aim was assessed by comparing group differences at 12-month follow-up under an intention to treat approach. First, the primary trial outcomes were compared between groups using all available data. For each primary outcome (proportion of schools with no ‘red’ and ‘banned’ items; and the proportion with >50% ‘green’ items), between-group differences were examined using Fisher’s exact test, and *p*-values and 95% confidence intervals for the relative difference in the proportion of schools complying with the policy were presented. This was also reported according to whether schools reported receiving other support to implement the policy. For the secondary outcomes of menu composition (percentage of ‘red’ and ‘green’ items on the menu), linear regression models were used to assess between-group differences at follow-up on the absolute scale (adjusting for the baseline value of the outcome), and corresponding Wald test *p*-values and 95% CIs are presented. Only schools that provided their menus and any additional information to the dietitian for menu review were included in this primary analysis. Sensitivity analyses was undertaken for all outcomes using multiple imputation methods to assess the impact of missing data as specified by White et al. for intention to treat analysis for trials with missing data [34]. Specifically, we used the chained equations method of generating complete datasets at random assumption. The imputation models were fitted separately by treatment group. We explored including a range of auxiliary school characteristics in the imputation model (including school socioeconomic status, education sector, type of Canteen Manager, days of operation, school size and number of volunteers). To reduce the risk of overfitting due to small sample size [35], these were not included as they did not appear to be associated with either the outcome or the missing status of the outcome. The Cochran Armitage test was used to assess the association between the number of audit and feedback cycles delivered (defined as one menu audit and provision of at least one modality of feedback i.e. written and/or verbal) and the proportion of schools with menus that had no ‘red’ and ‘banned’ items; or that had >50% ‘green’ items. Descriptive statistics were presented for the acceptability of each intervention component and type of policy implementation support received by schools.

### Sample size

Based on a previously published study [32] reporting a 15% compliance rate with the ‘Fresh Tastes @ School’ policy at follow-up in the control group, 72 schools (36 in the intervention and 36 in the control) with 80% power was calculated to enable detection of an absolute difference of 30% between groups in both primary outcomes, with a significance level of 0.05. An alpha of 0.05 was applied as it was anticipated that the primary outcomes would be correlated, as both relate to the food listed on canteen menus.

## Results

**Fig. 1:**
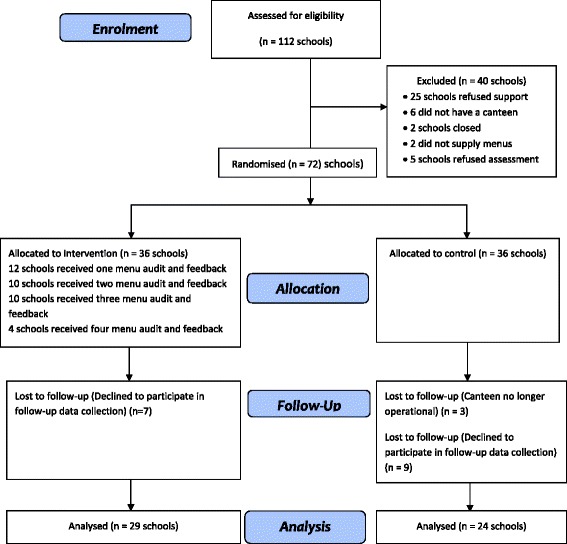
CONSORT diagram for primary schools enrolled in the trial

Of the rural and remote schools located within the study region, 150 schools that previously reported that they had a canteen were considered for eligibility, and 112 (75%) returned a menu for audit. Of those that did not return a menu, 30 schools did not consent to the audit, seven did not have a canteen, and one school had closed. Of the 112 schools that returned menus for audit, 72 (64%) consented to receiving support and were randomised to either the intervention or control group (36 in each arm – Fig. 1).

### School and canteen characteristics

No differences in the baseline characteristics of participating schools in the intervention and control group were apparent (see Table 1). All schools provided menus for baseline audit and 53 (74%) (29 intervention and 24 control schools) provided menus at follow-up. There were no significant differences in school characteristics between those that did and did not provide complete follow-up data (results not shown; *p* = 0.14–0.92).

**Table 1:** Baseline characteristics of participating schools by group

	Intervention *n* = 36	Control *n* = 36
Mean (SD) number of students	216 (176)	210 (219)
Socioeconomic Region^a^ (SEIFA 2006) n (%)		
Least advantaged	29 (81%)	33 (94%)
Most advantaged	7 (19%)	2 (5.7%)
School type n (%)		
Government	29 (81%)	29 (81%)
Catholic	7 (19%)	7 (19%)
Type of Manager^a^ n (%)		
Paid manager	16 (53%)	14 (45%)
Volunteer manager	13 (43%)	16 (52%)
Other	1 (3.3%)	1 (3.2%)
Days of operation^b^ n (%)		
5 days a week	13 (42%)	13 (42%)
3–4 days a week	4 (13%)	5 (16%)
1–2 days a week	14 (45%)	13 (42%)
Number of volunteers n (sd)	2 (1)	2 (2)

^a^Missing data from 1 control school

^b^Missing data from 11 schools (6 intervention; 5 control)

### Primary trial outcomes

At follow-up, in the analyses using all available data, the proportion of intervention schools without ‘red’ or ‘banned’ items on their menu, was not statistically significantly different from the proportion of control schools (35% for intervention, 13% control at follow-up; RR = 2.8; 95% CI: 0.9 to 8.9; *p* = 0.0895). The proportion of intervention schools that had more than 50% of items classified as ‘green’ was also not statistically significantly different to that of control schools (45% for intervention, 29% for control at follow up; RR = 1.5; 95% CI: 0.7 to 3.2; *p* = 0.2568) (see Table 2). Results remained non-significant but in the same direction for the multiple imputations analysis for both outcomes: i) having a menu with no ‘red’ or ‘banned’ items (RR: 2.31; 95% CI: 0.7, 7.2, *p* = 0.0907); ii) having a menu with more than 50% of items classified as ‘green’ (RR: 1.43; 95% CI: 0.7, 30.1); *p* = 0.3316).

**Table 2:** Impact of the intervention on schools’ compliance with the NSW Healthy School Canteen Policy (‘Fresh Tastes @ School’) (*n* = 53)

Variable	Baseline *n*(%)		Follow-up *n*(%)		Intervention v Control at Follow-up (complete data)	
	Intervention (*n* = 36)	Control (*n* = 36)	Intervention (*n* = 29)	Control (*n* = 24)	Relative risk (95%CI)	P-value
Menu does not contain any ‘red’ or ‘banned’ items *n* (%)	1 (2.8)	3 (8.3)	10 (34.5)	3 (12.5)	2.8 (0.9, 8.9)	0.0895
More than 50% of products listed on the canteen menu are ‘green’ items n (%)	7 (19.4)	7 (19.4)	13 (44.8)	7 (29.2)	1.5 (0.7, 3.2)	0.2568

### Secondary outcomes

#### Menu composition – percentage of green and red foods

Compared to controls, intervention schools were significantly more likely to have an absolute lower percentage of ‘red’ foods on the menu (6.0% in intervention, 8.8% in control; *p*-value: 0.007) and a higher percentage of ‘green’ items (47.9% in intervention, 38.0% in control; *p*-value: 0.014) (see Table 3). This effect was statistically significant in the multiple imputation analyses for the percentage of ‘red’ items (*p*-value: 0.0081) but not for the percentage of ‘green’ items on the menu (*p*-value: 0.0910).

**Table 3:** Impact of the intervention on percentage of ‘red’, ‘green’ and ‘amber’ foods on the menu controlling for baseline (*n* = 53)

Variable	Baseline n(%)		Follow up n(%)		Intervention v Control	
	Intervention (*n* = 36)	Control (*n* = 36)	Intervention (*n* = 29)	Control (*n* = 24)	Estimated difference (95% CI)	*P*-value
% ‘red’ on menu Mean (sd)	12.1 (8.4)	8.0 (9. 0)	6.0 (8.3)	8.8 (8.9)	−5.41 (−9.37, −1.45)	0.007
% ‘green’ on menu Mean (sd)	35.3 (15.3)	37.7 (15.7)	47.9 (16.0)	38.0 (18.5)	10.55 (2.06, 19.05)	0.014
% ‘amber’ on menu Mean (sd)	49.2 (12.3)	52.2 (14.7)	45.2 (13.6)	51.3 (15.0)	−4.63 (−11.82, 2.55)	0.206

#### Number of feedback reports and calls provided

Based on internal project management records, all schools received at least one menu audit and were provided with a written feedback report. Seventeen schools (53%) also received an initial feedback call. Twenty-three schools received two or more feedback reports (72%) and 21 schools (66%) received two or more feedback calls.

#### Perceived usefulness of intervention

Twenty-two Canteen Managers reported on the extent to which they found the intervention strategies helpful to support their implementation of the policy. The menu audit and feedback reports were rated as ‘very’ or ‘extremely helpful’ by 60.9% (*n =* 14) of Canteen Managers surveyed. About one third did not recall receiving the telephone support calls for delivery of verbal feedback (27.3%), while 40.9% rated the calls as ‘very’ or ‘extremely helpful’.

#### Receipt of other support to implement the policy

Of the Principals and Canteen Managers completing the telephone interview at follow-up, ten schools in the intervention group (34%) and eight schools in the control group (34%) reported receiving support from other organisations. Other programs that schools accessed include the Fresh for Kids program (four control and two intervention schools), the Stephanie Alexander Kitchen program (one control and one intervention school) and the Cancer Council Eat it To Beat it program (one control school). Thirty-six schools (18 control and 18 intervention schools) were also members of the Healthy Kids Association, which provides support to schools in NSW to meet the Healthy School Canteen Policy, and two intervention schools and two control schools reported receiving support from the organisation within the previous 12 months. A subgroup analyses by reported receipt of other implementation support indicated there was no difference in the proportion of schools with no ‘red’ foods (RR = 7.36 (95% CI: 0.45, 119.38), *p*-value: 0.1465), or in the proportion of schools with more than 50% ‘green’ foods on the menu (RR = 2.40 (95% CI: 0.65, 8.83), *p*-value: 0.2447).

#### A*ssociation between number of audit and feedback cycles and policy compliance*

A school was considered to have received a full cycle if they received a menu audit, a written feedback report and a feedback call. Of the intervention schools (*n* = 36), 12 schools (33%) received only one cycle, a further 10 schools received two cycles (28%), 10 schools (31%) received three cycles and four schools (13%) received four full cycles. The number of audit and feedback cycles provided was positively associated with a larger proportion of schools having a menu without ‘red’ or ‘banned’ items (*p* = 0.012) or having a menu where more than 50% of items were classified as ‘green’ (*p* = 0.0042). See Table 4.

**Table 4:** School compliance to NSW Healthy School Canteen Policy by number of audit and feedback cycle received

Outcome	Number Audit Feedback Cycles n (%)^a^				*p*-value
	1 (*n* = 12)	2 (*n* = 8)	3 (*n* = 6)	4 (*n* = 3)	
Menu does not contain any ‘red’ or ‘banned’ items n (%)	1 (8.3)	4 (50)	3 (50)	3 (100)	0.012^*^
More than 50% of products listed on the canteen menu are ‘green’ items n (%)	2 (17)	5 (63)	4 (67)	3 (100)	0.0042^**^

^*^ *p* < 0.05; ^**^ *p* < 0.001

^a^only 29 schools at follow up

## Discussion

The study found that a multicomponent audit and feedback intervention did not significantly increase the proportion of rural and remote schools within the region that had no ‘red’ foods or ‘banned’ drinks on their menu, or that had more than 50% ‘green’ items on their menus in accordance with the NSW ‘Fresh Tastes @ School’ Policy. Despite this, an absolute difference of 22% was achieved in the proportion of schools that had removed all ‘red’ and ‘banned’ items and this finding approached significance (*p* = 0.0895). There was also a significant change in the percentage of ‘red’ foods on the menus, suggesting that the intervention resulted in an positive change in menu composition in terms of a reduction in the availability of unhealthy foods. The complete-case analyses found that the intervention was significant in increasing the percentage of ‘green’ foods on the menu (*p* = 0.014). However in the multiple imputation analyses, this finding was no longer significant. While the use of multiple imputation increased the overall analytic sample size, it also introduced imputation variability to the study findings, as there were no variables that were strongly predictive of the outcome or missing status of the outcome. As such, the impact of the intervention on percentage ‘green’ foods on the menu remains uncertain.

The results of our study are comparable to the other implementation trial in this setting, which found no impact of a multicomponent intervention which included provision of training, financial support and resources on the nutrition composition of schools cafeteria meals [20]. While the current study achieved higher absolute effect sizes for the primary outcomes compared to the median effect size of 4.3% reported in audit and feedback interventions in clinical settings (interquartile range 0.5 to 16%) [26]), it was lower than the anticipated effect size specified for this study. We anticipated that this study would achieve a greater effect size compared to previous studies as the intervention protocol attempted to incorporate characteristics suggested to enhance the effectiveness of audit and feedback interventions. The intervention effect observed in our study could however have been diminished by a smaller than intended number of feedback rounds provided to schools across the intervention group. For example, while all schools received at least one menu audit and written report, only 72% of schools received two or more audit and feedback cycles. Systematic review evidence indicates that more than one contact is needed to maximise the effectiveness of audit and feedback interventions. Interestingly, findings from the Cochran-Armitage test found that increased intervention dose (i.e. number of audit and feedback cycles) was associated with increased policy compliance. Such findings are consistent with previous findings which report that more frequent feedback is likely to predict intervention effectiveness [26].

While the impact of audit and feedback on clinical practice has been extensively examined in other settings, this study is the first to examine the impact of such intervention in schools. Findings from this study suggest that audit and feedback interventions delivered in isolation may not be effective in improving school compliance with a healthy canteen policy. Despite findings of non-significance for the primary outcome, promising outcomes in relation to overall menu composition and the findings of an association between the number of cycles delivered and policy compliance suggest that future studies should explore reasons for lack of uptake of such interventions amongst school staff. Anecdotal discussions with Health Promotion Officers indicated that the key reasons for non-engagement was the time required by canteen managers to engage in the intervention and the multiple cycles of audit and feedback offered in a relatively short time frame. Incorporating strategies to support intervention uptake, such as reducing the time burden on Canteen Managers for the menu audit process and refining the number of audit and feedback cycles delivered, could increase the impact of future audit and feedback interventions on school nutrition policy compliance. Further, the majority of Canteen Managers reported finding the feedback reports ‘very’ or ‘extremely helpful’, suggesting that there is potential to reduce the number of feedback calls delivered as part of future interventions. While the current study was powered to detect a 30% difference in the primary outcomes, closer consideration of what constitutes a meaningful effect size for the setting is needed, given the potential health impact of implementation of healthy canteen policies on the diets of hundreds of thousands of children that attend NSW schools. Future trials could be powered to detect a smaller but clinically meaningful change given the potential for this intervention to be delivered to a large number of schools, across a wide geographical area.

### Limitations

A limitation of this study is that the intervention was not delivered as intended to all schools, with only 72% of schools receiving more than one round of the intervention. However, Health Promotion Officers responsible for delivering the intervention attempted to make multiple contacts with Canteen Managers and on most occasions were unable to deliver intervention support due to a lack of time or resistance from the Canteen Manager. The subgroup analyses examining exposure to other intervention support should be interpreted with caution given the small sample size. Future studies with larger sample sizes should pre-specify subgroup analyses by school characteristics (i.e. socioeconomic status, school size) to allow examination of whether intervention effects might differ by these subgroups. Another limitation is that the study had a moderate loss to follow up (30%). However, as our sensitivity analysis involving multiple imputation of missing data revealed similar results for the primary outcome, we have confidence in the study findings.

## Conclusions

Overall, this study found that a multicomponent audit and feedback intervention did not significantly improve rural and remote schools’ compliance with the NSW Healthy School Canteen Policy. The intervention was effective in reducing the number of ‘red’ items, suggesting an overall reduction in availability of unhealthy items. Future trials testing the impact of a more comprehensive range of strategies to improve the implementation of healthy canteen policies may be warranted given the lack of known effective implementation interventions in this setting.

## Abbreviations

ARIA: Accessibility/Remoteness Index of Australia
CATI: Computer Assisted Telephone Interview
HNELHD: Hunter New England Local Health District
NSW: New South Wales
RR: Relative risk
